# Nipocalimab‐aahu in generalised myasthenia gravis: A new Fc receptor‐targeted option for antibody‐positive patients

**DOI:** 10.1002/ibra.70006

**Published:** 2025-11-27

**Authors:** Jawairya Muhammad Hussain, Anoosha Ashfaq, Azka Shahid, Fatima Mudassir

**Affiliations:** ^1^ Department of Medicine Karachi Medical and Dental College Karachi Pakistan; ^2^ Department of Medicine Allama Iqbal Medical College Lahore Pakistan

**Keywords:** efficacy, FcRn receptor blockers, myasthenia gravis, Nipocalimab‐aahu

## Abstract

Myasthenia gravis (MG) is an autoimmune disorder driven by pathogenic IgG autoantibodies, leading to muscle weakness and impaired quality of life. Conventional immunosuppressant therapies are often limited by side effects and incomplete efficacy. Nipocalimab‐aahu (N‐a), recently FDA‐approved, is a novel neonatal Fc receptor (FcRn) antagonist that reduces circulating IgG levels, targeting the underlying disease mechanism. This review synthesizes evidence on N‐a's efficacy, safety, and clinical positioning. Phase 2 and 3 trials (Vivacity‐MG/MG3) demonstrated that N‐a significantly improves the Myasthenia Gravis Activities of Daily Living (MG‐ADL) score compared to placebo, with a favorable safety profile. The most common adverse events were nasopharyngitis and headache, with no increased serious infection risk or clinically significant hypoalbuminemia. Compared to other biologics, N‐a offers a distinct mechanism, selective IgG reduction without broad immunosuppression and a convenient bi‐weekly dosing regimen. While it shows promise for reduced systemic immunosuppression and potentially greater efficacy in MG‐ADL improvement than some alternatives, cross‐trial comparisons are limited. Key limitations include the need for long‐term safety data and more research in underrepresented populations, including pediatric and seronegative patients. In conclusion, N‐a represents a significant advancement in generalized MG (gMG) treatment, providing a targeted, effective, and well‐tolerated option that addresses a key unmet need for patients with acetylcholine receptor (AChR) or muscle‐specific kinase (MuSK) antibody‐positive disease.

## Graphical abstract

Generalised myasthenia gravis (gMG) can be effectively treated with nipocalimab (Imaavy), a new FcRn inhibitor that selectively lowers harmful IgG levels without causing widespread immunosuppression. According to Phase 2/3 trials, nipocalimab exhibits favourable safety, broad age applicability (≥12 years), and sustained efficacy, setting it apart as a promising therapeutic alternative with fewer side effects and better patient convenience than traditional immunosuppressants or complement inhibitors.

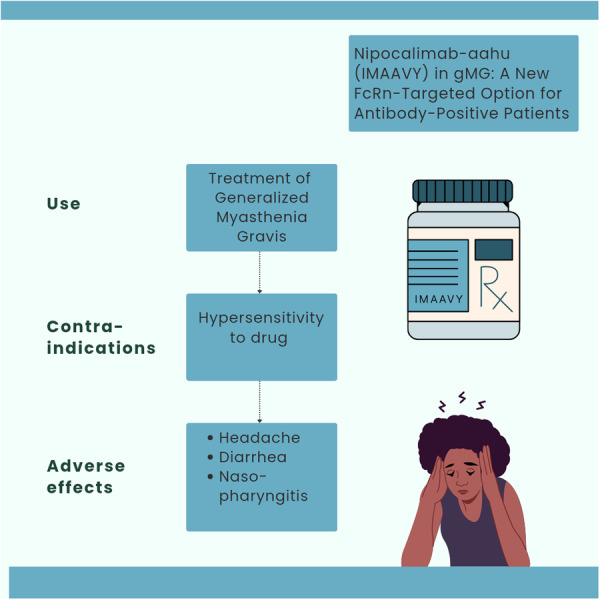

## 1 INTRODUCTION

Autoantibodies targeting postsynaptic receptors at the neuromuscular junction cause myasthenia gravis (MG), a chronic autoimmune disorder characterized by muscle weakness, persistent fatigue, and reduced quality of life. The incidence of MG ranges from 1.5 to 17.9 per 100,000 population, with prevalence estimates between 2.19 and 36.71 per 100,000, corresponding to approximately 56,000–123,000 patients in Europe and 60,000 in the United States.^1^ The Myasthenia Gravis Activities of Daily Living (MG‐ADL) score is a widely used measure of the functional impact of MG on patients' daily lives. Most patients require long‐term use of immunosuppressant drugs (ISDs), which are often associated with significant side effects and incomplete symptom control. There remains an unmet need for treatments that address the underlying disease mechanism while maintaining a favorable safety profile. The recent FDA approval of nipocalimab‐aahu (N‐a) (IMAAVY™) on April 29, 2025, represents an important advancement in this regard.^2^ The drug is administered by intravenous infusion, at least 30 min for the initial dose, followed by maintenance infusions every 2 weeks for at least 15 min.^2^ This paper highlights the efficacy of N‐a compared to conventional ISDs and discusses its potential as a targeted therapy for generalized MG (gMG) that offers reduced systemic immunosuppression and fewer adverse events (AE).

## 2 MECHANISM OF ACTION

N‐a acts by blocking the neonatal Fc receptor (FcRn) and is approved for patients aged 12 years or older who are positive for acetylcholine receptor (AChR) or muscle‐specific kinase (MuSK) antibodies. N‐a (M281) is an aglycosylated, effectorless, fully human IgG1 monoclonal antibody with high affinity for FcRn, effectively competing with IgG for its binding site. Under physiological conditions, FcRn recycles IgG antibodies, preventing their lysosomal degradation. N‐a disrupts this recycling, directing unbound IgG to lysosomal catabolism, thereby reducing circulating pathogenic autoantibodies in MG. This targeted approach minimizes broad immunosuppression while addressing the underlying pathogenic mechanism.^3^ It is contraindicated in patients with a history of serious hypersensitivity reaction to N‐a or to any of the excipients in it.

## 3 CLINICAL EFFICACY

### 3.1 Phase 2 evidence

In the Phase 2 Vivacity‐MG trial,^3^ infection and overall treatment‐emergent adverse events (TEAEs) rates were similar between the combined N‐a and placebo groups (33.3% vs. 21.4% and 83.3% vs. 78.6%, respectively). No serious AEs, including deaths, treatment discontinuations, grade ≥3 infections, or clinically significant laboratory abnormalities (e.g., hypoalbuminemia), were observed with N‐a. While lower doses did not yield statistically significant MG‐ADL improvements compared with placebo, a significant linear dose–response was observed (linear trend *p* = 0.03; rank‐based trend *p* = 0.004). The most consistent clinical responses were seen with 60 mg/kg single dose (*p* = 0.046) and 60 mg/kg every 2 weeks (*p* = 0.02), where 42.9%–64.3% of patients achieved clinically meaningful MG‐ADL improvements.^3^ Consistent with its mechanism of action, N‐a produced marked dose‐dependent reductions in total serum IgG (up to 83% at 60 mg/kg once every 2 weeks), accompanied by reductions in anti‐AChR autoantibodies and all IgG subtypes; patients with greater IgG reductions tended to show greater MG‐ADL improvements, supporting IgG lowering as a potential biomarker of efficacy. Immunogenicity was observed in ~40.7% of patients, but antidrug antibodies (ADA) were transient and did not affect pharmacokinetic, pharmacodynamics, efficacy, or safety outcomes.

While the trial provided encouraging signals of clinical benefit, it was not powered for formal pairwise efficacy comparisons, and findings should be regarded as hypothesis‐generating. The small sample size within each dosing arm, the short 8‐week treatment window, and study disruptions related to the COVID‐19 pandemic further limited the ability to detect consistent treatment effects across endpoints such as Quantitative Myasthenia Gravis (QMG). Subgroup analysis, including the apparent benefit of the 60 mg/kg dosing regimen, requires cautious interpretation given the exploratory nature of the analyses and potential sampling variability. To enhance clarity, the key phase 2 findings are summarized in Table 1.

**Table 1:** Key summary of efficacy and safety findings from phase 2 trial.

Endpoints	Findings	Statistical notes
Infections	Nipocalimab 33.3% vs. placebo 21.4%	Comparable rates
TEAEs	Nipocalimab 83.3% vs. placebo 78.6%	No grade ≥3 infections, no discontinuations
MG‐ADL (pairwise vs placebo)	*p* = 0.99, 0.24, 0.36, 0.22	None significant, confidence interval (CI) −3.5 to 3.1
MG‐ADL (dose‐response)	A significant linear dose–response was observed	Linear trend *p* = 0.03; rank‐based trend *p* = 0.004
Sustained MG‐ADL response	42.9–64.3% with 60 mg/kg regimen	*p* = 0.046 single dose, *p* = 0.02 Q2W
IgG Reduction	Up to 83% at 60 mg/kg Q2W, reduction in all subtypes	Correlated with MG‐ADL benefits

Abbreviations: MG‐ADL, Myasthenia Gravis Activities of Daily Living; Q2W, once every 2 weeks; TEAEs, treatment‐emergent adverse events.

### 3.2 Phase 3 evidence

In the Phase 3 Vivacity‐MG3 study, the least‐squares mean change in MG‐ADL score from baseline to weeks 22–24 was −4.70 in the N‐a group, compared with −3.25 in the placebo group. This difference reflects a statistically significant improvement in a substantial proportion of antibody‐positive patients with gMG, supporting N‐a's role as a safe option for prolonged disease control over 6 months when combined with standard‐of‐care therapies.^4^ The primary endpoint—mean change from baseline MG‐ADL score over Weeks 22–24, demonstrated statistical significance (95% CI: −2.38 to −0.52; *p* = 0.0024). The secondary endpoint, change in QMG score, was also significant (*p* < 0.001). Furthermore, the MG‐ADL responder rate (≥2‐point improvement over 22–24 weeks) reached statistical significance (*p* = 0.021), indicating that many patients achieved clinically meaningful benefits.^4^

While Vivacity‐MG3 confirmed significant improvements in MG‐ADL, QMG, and responder rates in the antibody‐positive population, certain interpretive limitations remain. Subgroup analyses, such as outcomes stratified by antibody status or prior therapy exposure, were exploratory and not specifically powered for definitive conclusions, meaning observed differences should be regarded as hypothesis‐generating. The trial duration of 24 weeks provides robust midterm efficacy and safety data, but longer follow‐up will be essential to assess the durability of benefit and the risk of rare AE. Additionally, although results were consistent across multiple endpoints and support N‐a as an effective and safe option, the study population may not fully represent all patients with gMG, warranting confirmation in broader clinical contexts.

## 4 COMPARISON WITH OTHER THERAPIES

Current gMG treatments include immunomodulators, ISDs, and cholinesterase inhibitors, which improve neuromuscular transmission. However, their long‐term use is limited by persistent symptoms, disease exacerbations, AE, and treatment intolerance. N‐a is well tolerated^3^ and approved for a broad age range of patients.^4^ In contrast, ravulizumab, a humanized recombinant IgG2/4κ monoclonal antibody, prevents membrane attack complex (MAC) formation by binding to complement component C5, thereby suppressing innate immune defense and increasing susceptibility to infection.^5^ N‐a, by selectively reducing IgG without affecting IgM, IgA, or IgE, avoids broad immunosuppression.^3^ Efgartigimod, another FcRn blocker, also reduces IgG but is not currently recommended for patients under 18; ongoing studies are evaluating its efficacy and safety in younger populations.^6^

Ravulizumab is administered intravenously using a weight‐based loading and maintenance schedule every 8 weeks.^7^ Efgartigimod is given at 10 mg/kg once weekly for 4 weeks in a treatment cycle.^6^ The FDA‐approved dosing regimen for N‐a is a 30 mg/kg intravenous loading dose, followed by 15 mg/kg every 2 weeks as maintenance.^4^ This makes N‐a potentially more convenient for patients compared with Efgartigimod, with less frequent dosing. In terms of safety, ravulizumab's C5 inhibition increases the risk of Neisseria meningitidis infection,^5^ while efgartigimod is most commonly associated with headache, upper respiratory tract infections, and urinary tract infections (≥10%).^6^ The most frequent AEs with N‐a were nasopharyngitis, headache, and diarrhea.^3^ Regarding efficacy, ravulizumab reduces MG‐ADL scores by approximately 1.7, efgartigimod by more than 2,^8^ and N‐a by more than 4.^4^ Across trials, anti‐FcRn agents (nipocalimab, efgartigimod) achieved significantly greater QMG score reductions compared with complement inhibitors such as ravulizumab (mean difference −2.60; 95% CI: −3.42 to −1.78; *p* < 0.001).^8^

**Figure 1:**
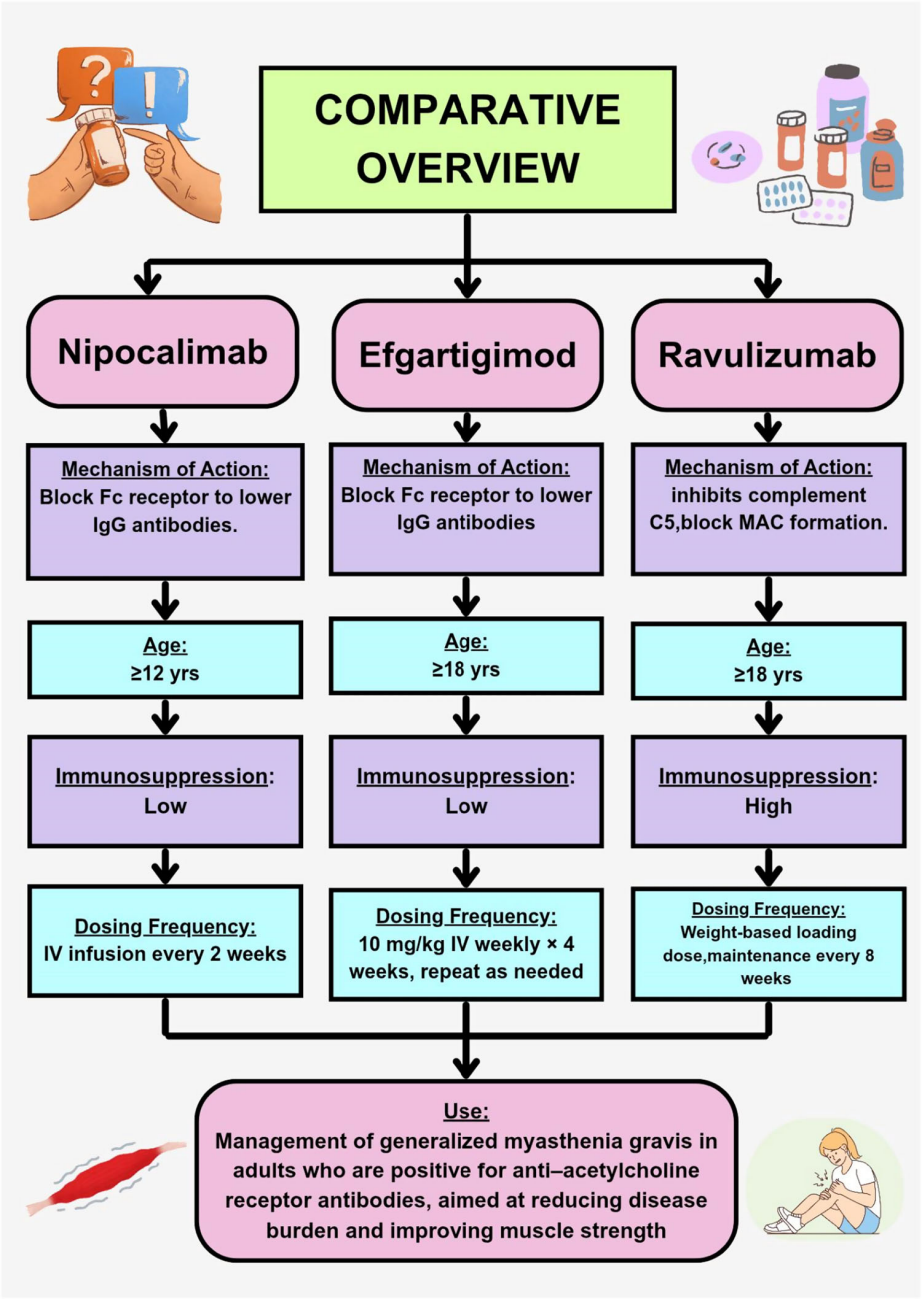
Comparative overview of therapies for generalized myasthenia gravis, highlighting differences in mechanism of action, age range, immunosuppression risk, and dosing frequency. IV, administered intravenously; MAC, membrane attack complex; yrs, years. [Color figure can be viewed at wileyonlinelibrary.com]

While the current evidence suggests differences in efficacy and safety profiles among N‐a, ravulizumab, and efgartigimod, it is important to interpret these comparisons with caution. Cross‐trial comparisons are inherently limited by heterogeneity in study designs, patient baseline characteristics, dosing regimens, and outcome measures. Such differences can impact observed efficacy and safety outcomes and preclude definitive conclusions regarding comparative effectiveness. Future head‐to‐head trials or well‐conducted indirect treatment comparisons are needed to more reliably assess the relative benefits and risks of these agents. The comparison of N‐a with other drugs is illustrated in Figure 1.

## 5 SAFETY PROFILE

Grade 3 AE of special interest (AESI) related to infections were not reported. By the end of the posttreatment follow‐up period, serum albumin levels had returned to baseline, despite the N‐a‐treated group exhibiting modest, dose‐dependent decreases in mean albumin levels. Throughout therapy, mean albumin concentrations remained within the normal range (3.5–5.5 g/dL), and no Grade 3 AESI due to hypoalbuminemia were observed. Mild, reversible, dose‐dependent increases were observed in low‐density lipoprotein (LDL), high‐density lipoprotein (HDL), and total cholesterol; however, no clinically significant changes in cholesterol levels were detected. Electrocardiograms, vital signs, physical examinations, and other laboratory assessments revealed no clinically significant abnormalities.^3^ In the Phase 3 trial, only one patient in the N‐a group (*n* = 98) experienced a serious, potentially life‐threatening AE, a myasthenic crisis.^4^

## 6 HEALTH ECONOMICS

The Vivacity‐MG3 study demonstrated sustained MG‐ADL improvements at Weeks 22–24; however, early time‐to‐response data and patient‐reported quality‐of‐life outcomes were not included in the primary publication. Supplementary materials suggest early (Week 2) improvements in EuroQol‐5D and a visual analogue scale (EQ‐5D VAS) scores and treatment satisfaction.^4^ Drug costs are up to $12,480 for a 1200 mg vial, making annual costs extremely high depending on the exact dosing regimen. However, with support programs for eligible patients, costs can be as low as $0 per infusion, which has the potential to make it accessible to a wider patient population.^9^ A post hoc analysis from Vivacity‐MG3 indicated that the therapy may reduce hospital and emergency department visits, potentially lowering annual healthcare costs by $8000–$10,000 per patient. While promising, this does not constitute a formal cost‐effectiveness evaluation; further pharmacoeconomic studies are required to assess broader healthcare and payer impact.^10^

To date, no published pharmacoeconomic analyses have specifically evaluated N‐a in gMG. Recent modeling presented at ISPOR 2024 for other FcRn inhibitors (efgartigimod and rozanolixizumab) estimated first‐year cost‐per‐responder values in the range of USD 360,000–400,000, underscoring the high economic stakes of these novel biologics.^11^ Whether N‐a will demonstrate comparable or differentiated cost‐effectiveness remains to be established through formal analyses. Importantly, real‐world value will likely vary across health systems, influenced by drug pricing negotiations, reimbursement frameworks, administration logistics, and regional willingness‐to‐pay thresholds. Thus, comprehensive cost‐utility and budget‐impact models that incorporate long‐term clinical outcomes and healthcare utilization are essential to inform payer decision‐making and equitable patient access.^11^

## 7 LIMITATIONS AND FUTURE DIRECTIONS

Despite their clinical potential, certain limitations remain for FcRn inhibitors, particularly N‐a. Evidence on their long‐term safety remains limited, as most available studies have involved small, homogeneous adult cohorts with short follow‐up durations, offering little insight into safety across diverse populations,^12^ including pregnant women and underrepresented ethnic groups. Seronegative and refractory patients were also underrepresented,^13^ limiting the generalizability of results. No clinical trials have yet evaluated the safety or efficacy of FcRn inhibitors in pediatric MG, and available evidence remains limited to adults aged ≥18 years. Similarly, data on seronegative MG, defined by the absence of detectable antibodies to AChR, MuSK, or low‐density lipoprotein receptor‐related protein 4 (LRP4), are sparse. Most FcRn inhibitor studies have enrolled exclusively seropositive patients.^14^ Although the underlying pathophysiology of seronegative MG is thought to be comparable and potentially responsive to FcRn blockade, this subgroup has been underrepresented in clinical research, and many reports do not explicitly state whether such patients were included. Recent reviews underscore the urgent need for dedicated trials to evaluate efficacy in this population.^14^

Addressing these gaps will require pediatric‐focused registries, detailed subgroup analyses, and population‐specific treatment strategies. Ensuring equitable access and inclusive representation in clinical guidelines will be essential for fully realizing the therapeutic potential of N‐a in gMG management. Future research should investigate FcRn inhibitors in combination with other immune‐targeting therapies to address multiple disease mechanisms.^14^ Trial designs must expand beyond generalized AChR‐positive MG to encompass other autoimmune disorders such as chronic inflammatory demyelinating polyneuropathy (CIDP), neuromyelitis optica spectrum disorders (NMOSD), stiff‐person syndrome, Guillain–Barré syndrome, and Sjögren's syndrome, while ensuring greater inclusion of underrepresented groups, including seronegative, refractory, and pediatric patients. Incorporating biomarkers into trial protocols may improve patient selection, optimize treatment timing, and enhance cost‐effectiveness.^13^ Head‐to‐head comparisons with other advanced therapies are also needed to guide optimal sequencing. Finally, studies should explore FcRn inhibitors as first‐line, steroid‐sparing agents, integrating patient‐reported outcomes to ensure alignment with patient priorities.

## 8 CONCLUSION

In conclusion, N‐a (IMAAVY™) represents a significant advancement in the treatment of gMG, offering targeted efficacy with fewer side effects and reduced immunosuppression. However, its broader clinical use warrants further evaluation, particularly regarding long‐term safety in underrepresented groups such as children, pregnant women, and diverse ethnic populations. Cost and access barriers also need to be addressed. With continued research and patient‐centered care, N‐a holds the potential to transform gMG management globally.

## AUTHOR CONTRIBUTIONS

Jawairya Muhammad Hussain conceived and led the project, curated the data, administered the project, created the visualizations, drafted the original manuscript, and revised it critically through review and editing. Anoosha Ashfaq participated in conceptualization, led the data curation process, and co‐drafted the original manuscript. Azka Shahid contributed to data curation and co‐authored the original draft. Fatima Mudassir was involved in data curation and co‐authored the original draft. All authors reviewed and approved the final version of the manuscript.

## CONFLICT OF INTEREST STATEMENT

The authors declare no conflicts of interest.

## ETHICS STATEMENT

Not applicable.

## DECLARATION ON THE USE OF AI

Generative AI tools (specifically ChatGPT) were used to assist with drafting and refining portions of the manuscript. These tools supported the writing process by helping improve clarity, language structure, and coherence across various sections. However, all scientific content, critical interpretation, and final revisions were conducted and verified by the authors to ensure accuracy and originality. The use of AI was limited to language enhancement and did not influence the scientific integrity or intellectual content of the article.

## Data Availability

Data sharing is not applicable to this article as no new data were created or analyzed in this study.
